# Exploring the Multifaceted Potential of Sildenafil in Medicine

**DOI:** 10.3390/medicina59122190

**Published:** 2023-12-17

**Authors:** Ciprian Pușcașu, Anca Zanfirescu, Simona Negreș, Oana Cristina Șeremet

**Affiliations:** Faculty of Pharmacy, “Carol Davila” University of Medicine and Pharmacy, Traian Vuia 6, 020956 Bucharest, Romania; ciprian.puscasu@umfcd.ro (C.P.); simona.negres@umfccd.ro (S.N.); oana.seremet@umfcd.ro (O.C.Ș.)

**Keywords:** sildenafil, drug repurposing, cancer, Alzheimer’s disease, depression

## Abstract

Phosphodiesterase type 5 (PDE5) is pivotal in cellular signalling, regulating cyclic guanosine monophosphate (cGMP) levels crucial for smooth muscle relaxation and vasodilation. By targeting cGMP for degradation, PDE5 inhibits sustained vasodilation. PDE5 operates in diverse anatomical regions, with its upregulation linked to various pathologies, including cancer and neurodegenerative diseases. Sildenafil, a selective PDE5 inhibitor, is prescribed for erectile dysfunction and pulmonary arterial hypertension. However, considering the extensive roles of PDE5, sildenafil might be useful in other pathologies. This review aims to comprehensively explore sildenafil’s therapeutic potential across medicine, addressing a gap in the current literature. Recognising sildenafil’s broader potential may unveil new treatment avenues, optimising existing approaches and broadening its clinical application.

## 1. Introduction

Sildenafil is the first oral medication approved by the United States Food and Drug Administration (FDA) for the therapeutic management of erectile dysfunction (ED) [1,2].

Originally developed with the intention of treating hypertension and angina pectoris, it showed no efficacy in phase 1 clinical studies, including patients with these pathologies. However, it induced a distinct pharmacological response in these individuals, inducing significant penile erection [1,3]. This accidental discovery led to the patenting of sildenafil by Pfizer in 1996, and, two years later, sildenafil received approval for the treatment of ED. Subsequently, several other uses of this pharmaceutical agent have been discovered [4].

**Figure 1:**
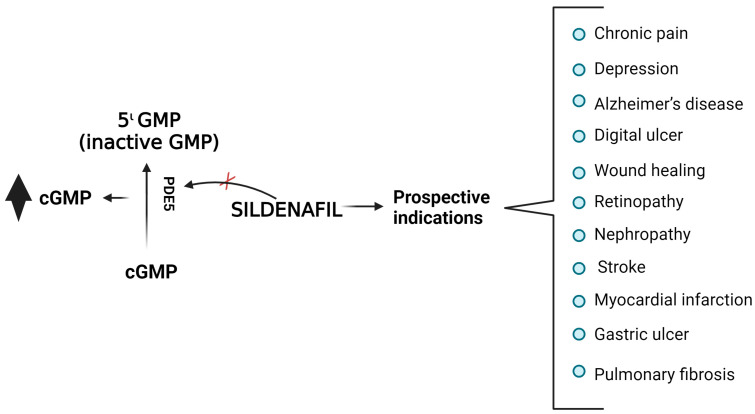
**The mechanism of action of sildenafil and its subsequent potential indications** (X-inhibition; 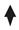 increased; created with BioRender version, Bio Rad 2023).

Sildenafil is a highly effective and specific inhibitor of phosphodiesterase type 5 (PDE5) [5,6]. There are several types of phosphodiesterases, but only three (PDE5, PDE6, and PDE9) specifically hydrolyse cyclic guanosine monophosphate (cGMP) over cyclic adenosine monophosphate (cAMP) [7,8]. It hydrolyses the phosphodiesterase linkage and facilitates the catalytic conversion of cGMP to inactive 5′-guanosine monophosphate (GMP), hence regulating several physiological processes within the body [9], such as neuroprotection, antinociception, synaptic plasticity, calcium homeostasis, and vasodilation (Figure 1) [10]. cGMP is involved in these processes through the activation of ion channels or cGMP-dependent protein kinases or through its interaction with PDE [9]. Nitric oxide (NO) promotes cGMP production in the target cell. NO and cGMP are integral constituents of a signalling transduction cascade that operates through autocrine, paracrine, and potentially endocrine mechanisms. The NO/cGMP/PDE5 axis has been implicated in various illnesses, including neurological disorders, pulmonary arterial hypertension, cardiomyopathy, cancer, ED, and lower urinary tract syndrome [8,11,12,13,14].

Additionally, sildenafil might possess other mechanisms of action and, consequently, supplementary therapeutic effects. Thus, sildenafil shows promising results in the treatment of neurodegenerative diseases through the activation of peroxisome proliferator-activated receptor-γ coactivator 1α (PGC1α), following the accumulation of cGMP, which increases mitochondrial biogenesis [15,16], upregulates antioxidant enzymes [17], and decreases β-site amyloid precursor protein-cleaving enzyme 1 (BACE1) expression [18]. 

Sildenafil also exhibits anti-inflammatory and neuroprotective properties, potentially mediated through the regulation of the AMP-activated protein kinase (AMPK)/nuclear factor of kappa light polypeptide gene enhancer in the B-cell inhibitor alpha (IKβα)/nuclear factor kappa B (NFκB) signalling pathway [19]. Furthermore, endothelial NO synthase (eNOS) contributes to the neuroprotective mechanism of sildenafil by promoting the activation of AMPK [19,20]. The potential of PDE5 inhibitors to treat chronic pain is supported by their ability to counteract the downregulation of angiopoietin 1 expression, a crucial factor involved in vascular stability and neurite development, within cultured dorsal root ganglion neurons [21,22].

Sildenafil might enhance the susceptibility of various types of tumour cells to the cytotoxic impact of chemotherapeutic agents [23]. This enhancement is achieved through the promotion of apoptosis, which is mediated by the downregulation of B-cell lymphoma-extralarge (Bcl-xL) and Fas-associated phosphatase-1 (FAP-1) expression, increased generation of reactive oxygen species (ROS), and upregulation of caspase-3, 8, and 9 activities [23,24,25]. 

The primary aim of this narrative review is to comprehensively investigate and synthesise the diverse therapeutic potentials of sildenafil within the field of medicine, as no such review currently exists. Understanding the broader potential of sildenafil can have implications for clinical practice. It may lead to the identification of new treatment options or the optimisation of existing ones, potentially expanding the range of conditions for which the drug could be considered. In summary, a review of the multifaceted potential of sildenafil in medicine serves as a valuable tool for consolidating existing knowledge, identifying new opportunities for research and clinical application, and potentially improving patient care.

## 2. Materials and Method

PUBMED was used to search the literature for the most relevant articles containing in vitro and in vivo preclinical and clinical findings on the effect of sildenafil in various conditions other than ED and PAH. We limited the search to articles published in English between 2015 and 2023. We did, however, investigate previous relevant research. We used the following keywords and MeSH terms: “sildenafil” AND “pain”, “sildenafil” AND “Alzheimer” OR “ Alzheimer disease”, “sildenafil” AND “ Raynaud’s” OR “Raynaud’s phenomenon”, “sildenafil” AND “digital ulcer”, “sildenafil” AND “wound healing”, “sildenafil” AND “cancer”, “sildenafil” AND “depression”, “sildenafil” AND “retinopathy”, “sildenafil” AND “renal” OR “renal disease” OR “kidney” OR “kidney disease” OR “nephropathy”, “sildenafil” AND “gastrointestinal” OR “gastrointestinal disease”, “sildenafil” AND “cardiovascular” OR “cardiovascular disease”, “sildenafil” AND “lung” OR “lung disease”. We selected the most appropriate studies after analysis and cross-checking. A literature study was also conducted to present the important characteristics of sildenafil pharmacokinetics, side effects, and current indications. We used the following keywords and MeSH terms: “sildenafil” AND “pharmacokinetics” OR “side effects” OR “indications” OR “erectile disfunction” OR “ED” OR “pulmonary arterial hypertension” OR “PAH”.

## 3. Sildenafil Pharmacokinetic Profile and Associated Adverse Reactions

Sildenafil citrate is rapidly absorbed from the gastrointestinal tract, reaching maximum plasma concentration approximately 30–120 min (with a median of 60 min) after oral administration. The absorption rate of sildenafil citrate is reduced when taken with food (particularly fatty foods) [26,27,28,29,30]. Along with its primary circulating N-desmethyl metabolite, sildenafil citrate exhibits a binding affinity of around 96% to plasma proteins [26,27,31]. It is mainly metabolised through the hepatic microsomal isoenzyme cytochrome P (CYP)3A4, with a slight contribution from the hepatic isoenzymes CYP2C9. Sildenafil citrate has 16 known metabolites. The primary metabolite, N-desmethyl sildenafil, exhibits a PDE selectivity profile comparable to that of sildenafil and an in vitro PDE5 potency roughly half that of the parent medication. The N-desmethyl metabolite is metabolised further, having a terminal half-life of about 4 h. Sildenafil citrate is a mild inhibitor of drug-metabolising enzymes, with CYP2C9 being the most inhibited [28,30,32]. Sildenafil citrate is mainly eliminated as metabolites in the faeces, accounting for approximately 80% of the orally taken dose. A smaller proportion is excreted in the urine, representing approximately 13% of the orally supplied dose. Its half-life ranges from 3 to 5 h [26,27,31].

The adverse effects of sildenafil are typically mild-to-moderate in severity and of a brief duration. Among the most frequent adverse reactions associated with the use of sildenafil are headaches, flushing, dyspepsia, and visual disturbances [31]. Mechanistically, these effects can be attributed to the vasodilatory properties of sildenafil. This vasodilation, although therapeutically beneficial, may cause headaches and flushing due to changes in blood flow. Additionally, altered retinal sensitivity to light may contribute to visual disturbances. Gastrointestinal effects, such as dyspepsia, may be linked to the presence of PDE5 in the smooth muscle of the gastrointestinal tract. Understanding these underlying mechanisms is crucial for both clinicians and researchers to optimise the therapeutic benefits of sildenafil while mitigating its associated adverse reactions [26,27,31]. Continued research in this area will undoubtedly refine our comprehension of the intricate pharmacological profile of sildenafil, further enhancing its clinical utility [26,27]. Sildenafil’s systemic vasodilation may result in a transient decrease in blood pressure, particularly when co-administered with nitrate-based medications. This hemodynamic effect underscores the importance of cautious prescribing, especially in patients with a history of myocardial infarction or unstable angina. It is crucial for healthcare providers to conduct a thorough cardiovascular assessment before prescribing sildenafil, ensuring a balanced consideration of its benefits against potential risks in individuals with underlying cardiovascular issues. Regular monitoring and personalised treatment plans remain integral in minimising the cardiovascular risks associated with sildenafil use [26,27].

## 4. Current Indications

Sildenafil is commonly utilised as a PDE5 inhibitor for two main indications.

### 4.1. Erectile Dysfunction (ED)

ED is a prevalent medical condition observed in males, and its frequency and occurrence rise with age. It is associated with diminished overall health and the coexistence of other medical conditions [33]. ED refers to the incapacity to attain or sustain an erection that is considered adequate for engaging in sexual activity [34]. The global occurrence of ED ranges from 4 to 66 cases per 1000 males per year [33,35,36,37,38]. In Europe, according to the European Male Ageing Study, ED prevalence ranges from 6% to 64% across various age groups. The prevalence of ED was found to increase with age, with an average prevalence of 30% [39]. Furthermore, the prevalence of ED appears to be higher in the United States, as well as in Eastern and Southeastern Asian countries, when compared to Europe or South America [40]. 

The NO–cGMP pathway serves as the principal mechanism for penile erection in primates, including humans. Sexual arousal activates neurological pathways, triggering the release of NO directly into the corpus cavernosum of the penis from both neurons and endothelial cells [11,41]. The biological stimulus responsible for initiating penile erection is cGMP, which then activates protein kinase G (PKG), responsible for the reduction of intracellular calcium levels. This reduction leads to the relaxation of arterial and trabecular smooth muscle, which subsequently causes arterial dilatation, venous constriction, and the attainment of penile erection stiffness [41,42]. PDE5 is the predominant PDE enzyme found in the corpus cavernosum, which facilitates the degradation of cGMP through the process of hydrolysis [9]. A reduction in NO levels may lead to ED [38].

The efficacy of sildenafil and other PDE5 inhibitors is widely recognised as the most successful approach for managing ED due to its high efficacy and remarkable lack of substantial adverse effects [43,44].

### 4.2. Pulmonary Arterial Hypertension (PAH)

PAH is characterised by elevated blood pressure in the pulmonary arteries. The walls of the pulmonary arteries become narrowed, thickened, or stiff, leading to increased resistance to blood flow. This elevated resistance eventually leads to right heart failure if left untreated. The Sixth World Symposium on PAH (2022) established a mean pulmonary artery pressure (mPAP) threshold exceeding 20 mmHg as the criterion for defining PAH [45]. The worldwide prevalence of PAH is significant, affecting around 1% of the global population [46].

There are various strategies for treating endothelial dysfunction. They usually aim to enhance the bioavailability of NO or the amounts of cGMP within cells. Subsequently, PDE5 inhibitors are frequently used for the management of PAH [42], being approved for its treatment in adults and children starting at the age of 1 year [8]. For PDE5 inhibitors to exhibit efficacy, it is imperative that there be an adequate level of activity within the NO/soluble guanylate cyclase (sGC)/cGMP pathway. In cases where NO is inadequately produced, an alternative approach should be used, such as sGC stimulators that are not dependent on NO [42].

The FDA specifically designates sildenafil for the therapeutic management of group 1 PAH [31], a chronic and progressive disorder characterised by the development of vascular abnormalities within the pulmonary circulation [46]. The addition of sildenafil to epoprostenol therapy can lead to a delay in clinical worsening in patients with New York Heart Association (NYHA) Functional Class II–III symptoms and idiopathic aetiology in 71% of cases and connective tissue disease in 25% of cases [47,48].

On the other hand, the European Medicines Agency (EMA) recommends the use of sildenafil for the treatment of adult patients diagnosed with PAH who are classed as WHO functional class II (slight limitation of physical activity) and III (marked limitation of physical activity) to enhance their exercise capacity [31]. Furthermore, EMA also designates sildenafil as a therapeutic option for the management of PAH in paediatric patients ranging from 1 to 17 years of age [31,49,50].

## 5. Prospective Indications

Drug repurposing continues to be a subject of great interest within the pharmaceutical and healthcare sectors, allowing the discovery of novel applications for medications licensed for other original indications [51]. Once a medicine was discovered to have an off-target or a newly recognised on-target effect, it was pushed forward for commercial exploitation [52].

As mentioned above, sildenafil is currently approved for the treatment of ED and PAH. Given the varying distribution of PDE5 across organs, recent research has extensively explored the potential therapeutic effect of sildenafil in various other conditions, such as pain, cancer, Alzheimer’s disease, depression, Raynaud’s phenomenon, digital ulcers, wound healing, retinopathy, gastric ulcer, colitis, nephropathy, lung injuries, and ischaemia, providing novel potential clinical applications for this molecule.

### 5.1. Pain

Local administration of L-arginine mitigates carrageenan-induced hyperalgesia, an effect prevented by NO inhibitors. Thus, cGMP was thought to be involved in the process of antinociception [53,54]. Subsequently, the analgesic effect of sildenafil was investigated using various animal models and dosages, showing a dose-dependent analgesic effect against diabetic and traumatic neuropathic pain, thermal hyperalgesia, and formalin-induced pain (Table 1). Intravenously administered sildenafil raises cGMP levels and, subsequently, modulates potassium channel function, promoting gamma-aminobutyric acid (GABA) release and resulting in an antinociceptive action. Thus, GABA receptors might mediate the pharmacological activity of sildenafil [55]. Additionally, PDE5 inhibitors seem to reduce chronic pain, mitigating the decrease in angiopoietin 1 expression, a critical regulator of blood vessel stability and nerve fibre growth in cultured dorsal root ganglion neurons [21,22,56].

### 5.2. Alzheimer Disease

Various sildenafil concentrations elicit the activation of PGC1α, triggering mitochondrial biogenesis [15,16], enhancing the production of antioxidant enzymes [17], and reducing BACE1 expression [18]. Thus, sildenafil might provide substantial advantages for individuals afflicted with Alzheimer’s disease. Furthermore, its vasodilatory effect might provide an additional benefit for individuals with Alzheimer’s disease since they exhibit cerebral hypoperfusion [63].

Sildenafil inhibits apoptosis in neurons experiencing hypoxia and facilitates neurogenesis [64,65,66,67,68]. Thus, it might decelerate the degeneration of neurons associated with Alzheimer’s disease and facilitate neurogenesis. Additionally, sildenafil enhances insulin sensitivity and reduces endothelial inflammation in individuals with diabetes. Therefore, it could potentially exert similar effects on insulin sensitivity and inflammation in the context of Alzheimer’s disease [69,70].

The in vitro administration of sildenafil protected brain mitochondria against β amyloid (Aβ) and advanced glycation end product (AGEPs)-induced injuries. In vivo, the administration of sildenafil induced an upregulation of brain-derived neurotrophic factor, a reduction in reactive astrocytes and microglia, a decrease in proinflammatory cytokines and neuronal apoptosis, and an increase in neurogenesis. Furthermore, in individuals with Alzheimer’s disease, sildenafil reduced aberrant spontaneous neural activity, increased cerebral blood flow, and enhanced the cerebral metabolic rate of oxygen (Table 2) [71,72].

### 5.3. Systemic Sclerosis-Associated Raynaud’s Disease and Digital Ulcer 

The pathophysiology of systemic sclerosis-induced vasculopathy includes reduced levels of NO. Subsequently, topical preparations that result in NO release induce a notable vasodilation in patients with this pathology [89,90]. The utilisation of a specific inhibitor targeting PDE5, such as sildenafil, is also an effective therapeutic approach for managing vascular disease in individuals with systemic sclerosis, considering that they have the ability to enhance the levels of NO and sustain peripheral blood circulation [91,92]. Clinical trials demonstrated that administration of sildenafil positively impacts the healing process of ischaemic digital ulcers in individuals diagnosed with systemic sclerosis, while in patients with Raynaud’s phenomenon, it decreases the severity, frequency, and duration of the disease (Table 3) [72,93,94].

### 5.4. Wound Healing

The treatment of chronic wounds continues to be a challenge in the medical field [107,108]. Tissue injury initiates a series of reparative processes aimed at restoring the structural integrity and functionality of the affected tissue [109], including enhancement of the clotting process, mitigation of oxidative stress, formation of new blood vessels, endothelial cell growth, and tissue remodelling [92]. NO seems to regulate some of these actions, leading to its recognition and utilisation as a biomarker for wound healing [110]. Moreover, deficiencies in inducible nitric oxide synthase (iNOS) and endothelial nitric oxide synthase (eNOS) have been linked to impaired wound healing [111,112]. Subsequently, sildenafil has been extensively studied in the field of wound healing, particularly in relation to its role in preserving the viability of skin flaps (Table 4).

### 5.5. Retinopathy

Other than its effects on PDE5, sildenafil also inhibits, to a smaller extent (about 10% of the activity of PDE5), PDE6, which is exclusively found in photoreceptor cells, being involved in phototransduction. Hence, it is plausible that the ocular manifestations associated with sildenafil use result from the suppression of PDE-6 activity [124,125].

PDE5 is found in the blood arteries of the choroid and retina [126]. Subsequently, sildenafil induces NO-induced vasodilation, particularly of the choroidal blood vessels, enhancing eye blood flow. This effect is probably achieved by the vasodilation process mediated by NO [127]. Furthermore, NO inhibits retinal vascular obliteration and the consequent development of proliferative retinopathies [128]. 

Animal studies demonstrated that sildenafil also reduced retinal vascular obliteration and neovascularisation. Additionally, it reduced the concentration of vascular endothelial growth factor (VEGF) in the ocular tissues, inhibited the progression of ischaemia injury, and reduced the thickness of the outer plexiform layer (Table 5). Thus, PDE5 inhibitors have the potential to impact disorders characterised by augmented choroidal thickness or choroidal and/or retinal ischaemia [129].

### 5.6. Cancer

PDE5 expression is elevated in various types of human carcinomas, such as urinary bladder tumours, metastatic breast cancers, and non-small cell lung cancers [133,134,135], possibly being involved in tumourigenesis. Hence, the suppression of PDE5 activity might result in antineoplastic properties [136].

Cyclic nucleotide second messengers, namely cGMP and cAMP, exhibit substrate affinity in the low micromolar range for ATP-binding cassette sub-family C member 4/multidrug resistance-associated protein 4 (ABCC4/MRP4) and ATP-binding cassette sub-family C member 5/multidrug resistance-associated protein 5 (ABCC5/MRP5). Sildenafil effectively suppresses the efflux function of ABCC4 and ABCC5 transporters [137,138]. Shi et al. demonstrated that sildenafil has inhibitory effects on ABC (ATP-binding cassette) transporters, specifically ABCB1 (ATP-binding cassette sub-family B) and ABCG2 (ATP-binding cassette sub-family G), and reverses multidrug resistance in cancer cells [139]. 

Numerous preclinical studies have documented the utilisation of sildenafil in conjunction with chemotherapeutic drugs for the management of diverse types of cancer (Table 6). Sildenafil has exhibited its efficacy in augmenting the delivery of anticancer drugs by leveraging the enhanced permeation retention (EPR) effect, leading to a substantial increase in drug concentrations within tumours and consequent induction of cellular apoptosis [23]. Furthermore, sildenafil has been shown to have a role in the regulation and enhancement of chemotherapeutic drugs in several cancer types. This phenomenon has been demonstrated in numerous in vitro and in vivo studies, wherein the downregulation of Bcl-xL expression; the promotion of ROS generation; the phosphorylation of BCL2-associated agonist of cell death (BAD) and B-cell lymphoma protein 2 (Bcl-2); the upregulation of caspase-3, 8, and 9 activities; the arrest of cells at the G0/G1 cell cycle phase; the circumvention of cancer cell resistance by inhibiting various ABC transporters through the elevation of cGMP; and the augmentation of autophagosome and autolysosome levels collectively induce apoptotic cell death in tumour cells [23,25,140].

In addition, clinical trials have been conducted to investigate the efficacy of sildenafil in the treatment of various forms of cancer (Table 6). The predominant focus of clinical research has been on the utilisation of sildenafil as a chemoadjuvant to improve its pharmacokinetic properties and to mitigate the adverse effects commonly associated with chemotherapy, specifically ED and cardiotoxicity.

### 5.7. Depression

Despite being primarily intended for peripheral tissue activity, sildenafil has the ability to traverse the blood–brain barrier (BBB) and impact central PDE5 activity, exhibiting various effects within the central nervous system, including the promotion of neurogenesis, augmentation of memory, mitigation of learning impairment, and neuroprotection [154]. Unsurprisingly, the utilisation of sildenafil to target PDE5 has emerged as a novel therapeutic approach in the management of various neurological and neuropsychiatric disorders [44], including depression. There is a strong correlation between depression and ED, with each condition mutually influencing and exacerbating the other. Consequently, failure may occur if one disease is treated while neglecting the other [155]. Additionally, ED can occur as an adverse effect of some antidepressant drugs [156]. Sexual dysfunction is also a prevalent symptom of major depressive disorder itself and may be linked to decreased levels of testosterone [157,158,159]. 

Clinical and preclinical studies have demonstrated that PDE5 inhibitors are effective not just in the treatment of ED, but also in ameliorating ED-associated depression [160]. Preclinical studies indicate an antidepressant effect similar to that of clinically used antidepressants. This action is believed to be mediated by the activation of oxytocin signalling pathways. Additionally, sildenafil increases the central concentrations of serotonin and noradrenaline [161,162] (Table 7).

### 5.8. Renal Diseases

Cyclic nucleotide signal transduction pathways are a burgeoning area of study in the field of kidney disease, with ongoing emphasis on the targeted inhibition of PDE5 [167]. Modulation of the cGMP-dependent protein kinase 1-phosphodiesterase (cGMP-cGK1-PDE) signalling pathway may be renoprotective and could lead to the development of innovative therapeutic approaches for chronic kidney disease. Subsequently, PDE5 inhibitors have been recognised as a prospective therapeutic alternative for various forms of renal injuries [168]. 

Multiple mechanisms have been postulated to play a role in counteracting the cascade of alterations generated by renal damage. The most extensively documented mechanisms by which PDE5 provides protection include stimulation of NO production via NOS activation, improvement of medullary blood flow, reversal of the Bcl2/Bax ratio, phosphorylation of extracellular signal-regulated kinase (ERK), activation of mitochondrial biogenesis, upregulation of renal progenitor cells, and regulation of multiple signalling pathways, including insulin/insulin-like growth factor 1 (IGF1) and nuclear factor NF-kB [169].

An elevation in ERK phosphorylation stimulates the activity of NOS, leading to an immediate release of NO [170]. In the days following renal injury, energy is required for the repair process, which is supplied by the cellular mitochondria. Despite the fact that mitochondria undergo continuous regeneration, cellular damage such as sepsis and hypoxia stimulates rapid biogenesis, and PGC-1α facilitates this process. PGC-1α induces the activation of nuclear respiratory factors 1 and 2, which subsequently stimulate mitochondrial transcription factor A, activating DNA (deoxyribonucleic acid) transcription within the mitochondria [16,171].

However, the protective effect of PDE5 operates via an alternative pathway. PDE5 increases cGMP, which stimulates PKG, which, in turn, opens mitochondrial KATP channels (ATP-sensitive potassium channel), causing Mg^2+^ release and depolarisation of the mitochondrial inner membrane. A depolarised membrane leads to a decrease in Ca^2+^ influx, which subsequently inhibits cellular death. An elevated concentration of Mg^2+^ decreases ROS and p38 mitogen-activated protein kinase (MAPK) activation, both of which are implicated in apoptosis [170,172,173].

Preclinical studies indicate that the administration of sildenafil improved renal function, decreasing the levels of oxidative stress indicators, such as malondialdehyde (MDA) and pro-inflammatory cytokines [174,175]. Furthermore, clinical studies further support this effect, that the administration of sildenafil decreases serum creatinine levels and increases the glomerular filtration rate. Additionally, sildenafil considerably reduces albuminuria, as well as haemoglobin A1c (HbA1c) levels (Table 8) [176,177].

### 5.9. Gastrointestinal Diseases

Gastric ulcers arise due to the harmful effects of acid and pepsin [206]. The stomach typically maintains a delicate equilibrium involving various components (such as defensive agents, the mucus bicarbonate barrier, surface epithelial cells, mucosal regeneration, blood circulation, acid–base balance, and epidermal growth factor) to counteract the effects of acid and pepsin present in the luminal content [207]. The equilibrium is typically disrupted due to Helicobacter pylori infection or prolonged use of nonsteroidal anti-inflammatory medicines (NSAIDs) [208]. NO exerts a substantial impact on the generation of mucosal resistance and repair [209], effectively safeguarding against stomach mucosal injury [210]. Consequently, sildenafil facilitates ulcer repair. 

Inflammatory bowel diseases, such as ulcerative colitis, are chronic conditions of unknown origin characterised by significant inflammation of the digestive tract [211]. Their pathogenesis comprises a complex interplay among genetic, immunological, and environmental variables. During the acute episodes of inflammation, there is an increase in inflammatory cells at the intestinal level, leading to excessive synthesis of proinflammatory mediators, including cytokines, eicosanoids, ROS, and nitrogen metabolites [212,213,214]. Sildenafil exerts anti-inflammatory effects by preventing lipid peroxidation, cytokine release, and oxidant generation [215,216].

Preclinical studies examined the therapeutic potential of sildenafil against various gastrointestinal diseases (Table 9), demonstrating that it effectively prevents ulceration by increasing the concentration of NO within the gastric tissue. Additionally, sildenafil enhances levels of antioxidant enzymes, reduces gastric acid secretion, decreases levels of proinflammatory cytokines, and increases the level of glutathione (GSH) [72,216,217].

### 5.10. Cardiovascular Diseases 

Extensive studies have been conducted on the possible application of sildenafil in the field of cardiovascular disease. Seven of the eleven major PDE subtypes—PDE1, 2, 3, 4, 5, 8, and 9—are expressed in the heart, and their diverse effects on cAMP and cGMP signalling in distinct cell types, including cardiomyocytes, provide therapeutic prospects to combat heart disease [226]. PDE5 is abundantly expressed in isolated canine or murine ventricular cardiomyocytes, and it is strongly expressed in both experimental and human heart disease. PDE5 inhibition should have the potential to alter heart function, contributing to the treatment of a range of cardiovascular illnesses [42].

The vasodilatory effects of PDE5 inhibitors have positive effects on vascular coagulopathy and enhance endothelial functioning in various regions of the body [227]. The presence of PDE5 antibodies has been detected in coronary vascular smooth muscle cells, it seems that healthy myocardium does not exhibit significant expression of this enzyme [8]. Nonetheless, in cases of congestive heart failure and right ventricular hypertrophy, the activation of angiotensin II in vascular smooth muscle cells results in an increase in PDE5 levels, thereby leading to a decrease in cGMP/PKG reactivity [228].

A strong correlation has been established between ED and endothelial dysfunction, indicating that ED might serve as an external indicator for various underlying cardiovascular diseases. Sildenafil enhances endothelial function in medical disorders such as diabetes and congestive heart failure and enhances perfusion and oxygen tension within the vasculature holding potential for addressing ischaemia/reperfusion injury, including myocardial infarction and stroke [1,229,230,231].

Preclinical and clinical trials demonstrate the cardioprotective effects associated with the inhibition of PDE5 in individuals diagnosed with established cardiovascular disease. In animal models, sildenafil enhanced both systolic and diastolic cardiac function, reduced cardiac hypertrophy and apoptosis of cardiomyocytes, and lowered the susceptibility to post-ischaemic arrhythmia [72,232,233]. In individuals diagnosed with heart failure, the administration of sildenafil decreased pulmonary artery pressure and alleviated dyspnoea. Additionally, it improved brachial artery flow-mediated dilatation and boosted breathing during physical exertion [234]. Moreover, in patients diagnosed with chronic stable left ventricular failure, sildenafil has demonstrated improvements in left ventricular ejection fraction and performance on the 6 min walking test (Table 10) [235].

### 5.11. Lung Diseases

While sildenafil has received approval for the treatment of PAH, studies have demonstrated its positive effects on various respiratory conditions, such as bronchopulmonary dysplasia and cystic fibrosis, while other conditions, such as asthma, had a negative impact. Furthermore, research conducted on animals has demonstrated that the administration of sildenafil in the treatment of PAH is associated with a decrease in several inflammatory mediators and the regulation of several intracellular signalling molecules, including MAPK, NF-kβ, and ERK [267]. Preclinical and clinical research indicates that sildenafil could potentially serve as a novel therapeutic approach for lung disorders, particularly those characterised by an inflammatory reaction (Table 11).

## 6. Summary

Our narrative review focused on summarising the potential clinical applications of sildenafil. Sildenafil, recognised for its vasodilatory effects, has shown potential therapeutic applications beyond ED and PAH, extending into various medical domains. In the realm of pain management, studies suggest that sildenafil may modulate nociceptive pathways. Its ability to increase pain reaction latency in animal models with neuropathic pain, such as those induced by diabetes, indicates a potential role in alleviating chronic pain [22,58]. The underlying mechanism involves the activation of the cGMP pathway, which is implicated in pain signal processing. Sildenafil’s influence on this pathway may contribute to the attenuation of pain responses, providing a foundation for exploring its utility in clinical settings addressing chronic pain conditions [53,54].

Emerging evidence also points to the neuroprotective effects of sildenafil in neurodegenerative diseases such as Alzheimer’s. The drug’s impact on cerebral blood flow and its ability to reduce oxidative stress and inflammation in the brain suggest potential benefits in mitigating neurodegenerative processes. The modulation of cGMP-dependent pathways and enhancement of neurotrophic factors are proposed mechanisms through which sildenafil may exert neuroprotective effects. This opens avenues for research into the use of sildenafil as a potential adjunctive therapy for neurodegenerative disorders [69,77,88].

In the context of systemic sclerosis-associated Raynaud’s disease and digital ulcers, sildenafil’s vasodilatory properties become particularly relevant. By inhibiting PDE5, the enzyme responsible for cGMP degradation, sildenafil prolongs the vasodilatory effects of NO. This mechanism improves blood flow, offering potential relief for patients with systemic sclerosis where vascular complications are prominent [91,92]. The positive impact on digital ulcers, a common complication in systemic sclerosis, suggests a role for sildenafil in managing the vascular manifestations of autoimmune diseases. The precise mechanisms involve enhanced vasodilation and improved microcirculation, showcasing the multifaceted therapeutic potential of sildenafil in systemic sclerosis-associated complications [72,93,94].

In the realm of cancer, studies propose that sildenafil’s inhibition of PDE5 could impact tumour growth [136]. Modulation of the cGMP signalling pathway influences angiogenesis and immune responses, enhancing the efficacy of chemotherapy and presenting a potential avenue for adjunctive cancer therapy [23]. In depression, sildenafil’s neuroprotective effects and its influence on neurotransmitter systems, including serotonin and dopamine, have been explored [154,161]. By enhancing cGMP signalling in the brain, sildenafil may exhibit antidepressant effects, paving the way for further investigations into its role in mood disorders.

Renal diseases, marked by impaired blood flow and inflammation, may benefit from sildenafil’s vasodilatory and anti-inflammatory properties. The drug’s ability to enhance renal blood flow through the relaxation of vascular smooth muscle cells offers a potential therapeutic avenue for conditions such as chronic kidney disease [169,202]. Gastrointestinal diseases, particularly those involving impaired blood perfusion, could also be targeted by sildenafil. The drug’s vasodilatory effects may aid in improving blood flow to the gastrointestinal tract, potentially mitigating complications in conditions such as inflammatory bowel disease [215,216]. Additionally, sildenafil’s impact on ischaemia/reperfusion injury holds promise in cardiovascular events such as myocardial infarction and stroke. The vasodilation induced by sildenafil can enhance blood flow during reperfusion, potentially reducing tissue damage and inflammation [1,229,230,231,244]. These multifaceted applications of sildenafil underscore its potential as a versatile therapeutic agent beyond its well-established roles, necessitating further research to unravel the full extent of its clinical utility.

While preclinical studies have demonstrated promising outcomes, including mechanisms of action and potential benefits, several limitations impede a straightforward transition to clinical applications. Preclinical trials often involve animal models, which may not fully mirror the complexities of human physiology and pathology. Additionally, variations in dosages, administration methods, and experimental designs among preclinical studies contribute to a lack of standardised protocols. Furthermore, the diverse nature of human diseases requires careful consideration of factors such as patient variability, comorbidities, and long-term effects, which may not be fully captured in preclinical settings. 

Supplementary limitations in the current body of research include a potential bias towards positive outcomes in published studies, which may not reflect the entire spectrum of findings and could skew the overall perception of sildenafil’s efficacy. Additionally, there is a need for more comprehensive investigations into potential adverse effects and interactions with other medications, especially in the context of chronic usage. Addressing these limitations is crucial for establishing the safety and effectiveness of sildenafil in diverse clinical scenarios.

Consequently, the current gap between preclinical evidence and clinical trials underscores the need for rigorous, well-designed clinical studies to validate the safety, efficacy, and optimal dosages of sildenafil in these diverse medical indications. Bridging this gap is crucial to ascertain the true therapeutic potential of sildenafil and to establish evidence-based guidelines for its application in clinical settings.

## 7. Conclusions

In conclusion, our comprehensive review highlights the diverse and promising therapeutic potential of sildenafil beyond its well-established uses. From its role in pain management, neurodegenerative diseases, and systemic sclerosis-associated complications to its impact on cancer, depression, and various organ-specific diseases, sildenafil emerges as a multifaceted therapeutic agent. However, rigorous, well-designed clinical studies are essential to guide evidence-based guidelines for the nuanced application of sildenafil in diverse clinical settings, ensuring its potential as a versatile therapeutic agent is realised to its fullest extent.

## Figures and Tables

**Table 1:** Preclinical studies that evaluated the effect of sildenafil on pain.

Pain			
Animals	Animal Model	Dosage	Results
db/db mice	Diabetic mice (type 2 diabetes)	10 mg/kg bw, orally	Sildenafil increased blood vessel functionality, regional blood flow in the sciatic nerve, and motor and sensory conduction velocities in the sciatic nerve, as well as ameliorated the sensitivity to peripheral heat stimuli in the tail flick test. [57]
NMRI male mice	Alloxan-induced diabetic neuropathy	1.5, 2.5, 3 mg/kg bw, orally	Sildenafil increased the pain reaction latency in both hot-plate tests and tail-withdrawal tests from cold water [58].
NMRI male mice	Alloxan-induced diabetic neuropathy	Sildenafil 1.5, 2.5, 3 mg/kg bw + Metformin 150, 250, 500 mg/kg bw, orally	The combination reversed thermal hyperalgesia in hot-plate and tail-withdrawal tests. It reduced the activity of iNOS in the brain and liver and IL-6 brain concentration. [22]
Wistar female rats	Formalin-induced pain	50, 100, and 200 µg into the ipsilateral paw 20 min before formalin	Locally administered sildenafil resulted in dose-dependent antinociception through the activation of the cyclic GMP-PKG-K+ channel pathway [59].
Wistar female rats	Formalin-induced pain	50, 100, and 200 µg, i.pl.	Sildenafil exhibited a dose-dependent antinociceptive effect and enhanced morphine-induced analgesia [53].
Wistar rats Albino mice	Carrageenan-induced hyperalgesia Acetic acid-induced pain	1, 2, 5, and 10 mg/kg, i.p. 50–200 µg, i.pl.	Administered i.p., sildenafil reduced acetic acid-induced writhing in mice in a dose-dependent manner. Local administration of sildenafil reduced the intensity of hyperalgesia induced by carrageenan [60].
Wistar rats Albino mice	Streptozotocin-induced diabetic neuropathy	1–10 mg/kg, i.p 50–400 µg/paw, i.pl.	Sildenafil increased the pain reaction latency in writhing tests (mice) and paw hyperalgesia tests (rats). [61].
Wistar male rats	Streptozotocin-induced diabetic neuropathy	5 mg/kg bw, orally	Sildenafil effectively and consistently enhanced vascular function both in terms of immediate and long-term effects [62].
Sprague–Dawley male rats	Ligation of L5/6 spinal nerves induced neuropathic pain	1, 3, 10, and 30 mg/kg bw i.p.	Sildenafil increased the withdrawal threshold in von Frey tests in a dose-dependent manner [55].

bw, body weight; GMP-PKG, cGMP-dependent protein kinase G; i.pl., intraplantar; IL6, interleukin 6; iNOS, inducible nitric oxide synthase.

**Table 2:** Preclinical and clinical studies that evaluated the effect of sildenafil on Alzheimer’s disease.

Alzheimer’s Disease		
In Vitro Studies		
Population	Dosage	Results
HT-22 mouse hippocampal neuronal cells treated with Aβ peptide	10–100 μM	Protects the mitochondria of neuronal cells from Aβ-induced injury. This effect is dependent on mitochondrial KATP channels. [73]
HT-22 mouse hippocampal neuronal cells exposed to advanced glycation end products	20 μM	Reduced the opening of mitochondrial permeability transition pores and HO1 induced apoptosis. [74]
Preclinical studies		
PS1/APP mice	3 mg/kg bw, i.p.	Enhanced immediate and prolonged synaptic function, phosphorylation of CREB, and memory; decreased Aß concentrations [75].
PS1/APP mice	10 mg/kg bw, i.p	Effectively mitigated memory impairments, restored the proper functioning of the cGMP/PKG/pCREB signalling pathway, and decreased Aβ1-40, Aβ1-42, IL-1β, IL-6, and TNF-α [76].
PS1/APP mice	6 mg/kg bw, i.p.	Enhanced memory, reduced amyloid plaque accumulation, mitigated inflammatory processes, and promoted neurogenesis [77].
PS1/APP mice	2 mg/kg bw, orally	Ameliorated memory deficits, reduced amyloid pathology, and increased the NOS, NO, and cGMP [78].
Sprague–Dawley rats with scopolamine-induced cholinergic dysfunction	1.5, 3.0, 4.5 mg/kg bw, i.p.	Demonstrated beneficial effect on memory Retrieval. [79].
Tg2576 transgenic mice	15 mg/kg bw, i.p.	Enhanced memory, reduced tau protein levels, inhibited the activity of GSK3β, lowered the CDK5 p25/35 ratio, and upregulated the expression of BDNF and Arc proteins [80].
J20 mice	15 mg/kg bw, in drinking water	Enhanced memory function, reduced tau hyperphosphorylation, and increased GSK3β-mediated phosphorylation of Akt [81].
C57Bl/6J WT mice	3 mg/kg bw, i.p.	Reduced double-stranded DNA breaks, downregulated pro-apoptotic factors caspase-3 and Bax, and upregulated anti-apoptotic factors Bcl2 and BDNF [82].
Aged male Fisher 344 rats	1.5, 3.0, 4.5, 10.0 mg/kg bw, i.p.	Enhanced spatial memory [83].
SAMP8 mice	7.5 mg/kg bw, i.p.	Enhanced amyloid and tau pathology, memory function, and gliosis [84].
SAMP8 mice	7.5 mg/kg bw, i.p.	Reduced hippocampal JNK phosphorylation and tau phosphorylation, as well as ameliorated memory impairments [85].
Sprague–Dawley male rats with aluminium-induced Alzheimer’s	15 mg/kg bw, orally	Reduced the expression of VCAM-1, TNF-α, oxidative stress markers, and α-synuclein immunostaining, while increasing the levels of VEGF-A and nestin [86].
Clinical studies		
Alzheimer’s disease (n = 10)	50 mg, orally	Reduced the intrinsic neuronal activity in the right hippocampus [87].
Alzheimer’s disease (n = 14)	50 mg, orally	Enhanced the cerebral metabolic rate of oxygen and cerebral blood flow (n = 12). Reduced cerebral vascular reactivity (n = 8) [88].

Arc protein, neuronal activity-induced expression of the immediate early protein; Aβ1-40, amyloid β-protein 1-40; Aβ1-42, amyloid β-protein 1-42; Bax, B-cell lymphoma protein 2 (Bcl-2)-associated X; Bcl2, B-cell lymphoma 2; BDNF, brain-derived neurotrophic factor; bw, body weight, CDK5 p25/35, cyclin-dependent kinase 5; cGMP, cyclic, guanosine monophosphate; CREB, AMP response element-binding protein; GSK3β, glycogen synthase kinase-3 beta; HO1, heme oxygenase 1; i.p., intraperitoneally; IL-1β, interleukin 1ß; IL-6, interleukin 6; JNK, Jun N-terminal kinases; NO, nitric oxyde; NOS, nitric oxide synthase; pCREB, phosphorylated transcription factor cAMP-response element-binding protein; PKG, protein kinase G; TNF-α, tumour necrosis factor α; VCAM-1, vascular cell adhesion molecule 1; VEGF-A, vascular endothelial growth factor A.

**Table 3:** Clinical studies evaluating the effect of sildenafil in digital ulcers and Raynaud’s syndrome.

Digital Ulcer		
Clinical Studies		
Population	Sildenafil Dosage (Oral Administration)	Results/Reference
Patients with systemic sclerosis and digital ulcers (n = 19)	114 mg	Reduces the quantity of digital ulcers (initial: 49, final: 17) [95].
Females with systemic sclerosis and digital ulcers (n = 1)	Sildenafil 20 mg + Bosentan 125 mg	Successfully heals pre-existing digital ulcers [96].
Patients with scleroderma (n = 10)	12.5 to 100 mg	Determines the complete resolution of digital ulcerations for eight individuals [97].
Patients with systemic sclerosis and digital ulcers (n = 6)	50 mg	Full healing (n = 2) or gradual improvement over time (n = 4) [93].
Patients with systemic sclerosis (n = 83) and digital ulcers (n = 192) Phase 3 study	20 mg	Significant reduction in the incidence of digital ulcers vs. placebo [98].
Raynaud’s disease		
Clinical studies		
Population	Dosage	Results
Symptomatic secondary Raynaud’s phenomenon (n = 16)	50 mg, orally	Decreases the average number and cumulative duration of Raynaud’s attacks. Decreases in the mean Raynaud’s Condition Score were observed. Significantly increased capillary blood flow [93].
Raynaud’s phenomenon secondary to systemic sclerosis (n = 57)	100 mg, orally	Reduced the frequency of attacks among patients, while exhibiting a high level of tolerability [94].
Raynaud’s phenomenon secondary to systemic sclerosis (n = 10)	50 mg, orally	Rapidly decreased the frequency and intensity of Raynaud’s phenomenon symptoms (n = 8) [97].
Raynaud’s phenomenon secondary to systemic sclerosis (n = 123)	Sildenafil 20 mg + Bosentan 125 mg, orally	Significantly enhanced Raynaud’s Condition Score and improved nailfold videocapillaroscopy [99].
Raynaud’s disease and joint pain (n = 1)	50 mg, orally	Induces vasodilation by upregulating cGMP and increasing NO synthesis [100].
Severe Raynaud’s phenomenon and systemic scleroderma	50 mg, orally	Significantly increased peripheral blood circulation and reduced the intensity and occurrence of Raynaud’s syndrome [101].
Primary Raynaud’ss disease (n = 15)	100 mg, orally	Increased the cutaneous vascular conductance and skin temperature [102].
Scleroderma-associated Raynaud’s phenomenon (n = 1)	50 mg, orally	Raised the peripheral blood perfusion and ameliorated the manifestation of Raynaud’s syndrome symptoms [103].
Severe Raynaud’s phenomenon and systemic sclerosis (n = 30)	Sildenafil 20 mg + Bosentan 125 mg, orally	Reduced the intensity, frequency, and duration of Raynaud’s phenomenon [96].
Severe Raynaud’s phenomenon associated with scleroderma (n = 1)	20 mg, orally	Enhanced the blood supply of microvasculature to the extremities and effectively mitigated the vascular alterations observed in Raynaud’s syndrome [104].
Secondary Raynaud’s phenomenon associated with connective tissue disease (n = 10) Phase 1 study	5 g of 5% cream	Significantly enhanced the digital artery blood flow [105].
Raynaud’s phenomenon secondary to systemic sclerosis (n = 41) Phase 3 study	100 mg, orally	Enhanced peripheral blood circulation and alleviation of symptoms associated with Raynaud’s phenomenon [106].

cGMP, cyclic guanosine monophosphate; NO, nitric oxide.

**Table 4:** Preclinical studies evaluating the effect of sildenafil in wound healing.

Wound Healing		
Preclinical Studies		
Population	Dosage	Results
Wistar rats with incisions of the dorsal backs	10 mg/kg bw, orally	The extent of skin flap necrosis was smaller in the sildenafil-treated groups than in the control group, albeit without statistical significance [113].
Sprague–Dawley rats with incisions of the dorsal backs	9 mg/kg, i.p.	A notable reduction in dead tissue and blood flow stagnation within the flap was observed in rats treated with sildenafil [114].
Wistar rats with incisions of the dorsal backs	10 mg/kg bw, orally	Increased the vascularisation of the skin flap [115].
Streptozotocin-induced diabetic Wistar rats with incisions of the dorsal backs	5% sildenafil-containing ointments	Significantly decreased wound area in both non-diabetic and diabetic rats, especially during the first stages of wound healing [116].
Wistar female rats with incisions of the abdominal wall	10 mg/kg, orally	Enhanced both the breaking strength of the abdominal fascia and the process of neovascularisation [117].
Sprague–Dawley female rats with incisions of the dorsal backs	0.4 g (1% gel) and 2 g (5% gel)	Enhanced vascularity, reduced inflammation, granulation tissue development, and maturation in a dose-dependent manner [118].
Sprague–Dawley male rats with incisions of the dorsal backs	10% hydrogel	Enhanced wound healing, reducing proinflammatory cytokine (IL-6, TNF-α, and IL-1β) levels as well as CRP levels. Increased the levels of tissue hydroxyproline, collagen, nitrite, and total protein content [119].
Wistar rats with incisions of the dorsal backs	10 mg/kg bw, orally	Decreased the size of full thickness defects [120].
Sprague–Dawley female rats with non-splinted excisions dorsal wounds	3, 5 and 10% hydrogel	Facilitated the process of reepithelisation, collagen synthesis, deposition, and regeneration of skin appendages [121].
Streptozotocin-induced diabetic Sprague–Dawley rats with incisions of the dorsal backs	0.7 mg/kg bw, i.p.	Regulated cellular activity during the initial stages of wound healing reducing lymphocyte numbers and increasing monocytes [122].
Streptozotocin-induced diabetic Wistar rats with incisions of the dorsal backs	10 mg/kg bw, orally	Inhibited the diabetic ulcerative process, reduced pain perception, and promoted wound healing [123].
Cross-breed street dogs with incisions in all skin layers on the anterior brachial region	25 mg	Increased the development of granulosa tissue and capillary network; enhanced fibroblast proliferation [109].

bw, body weight; CRP, C-reactive protein; i.p., intraperitoneally; IL-1β, interleukin 1ß; IL-6, interleukin 6; TNF-α, tumour necrosis factor α.

**Table 5:** Preclinical studies evaluating the benefits of sildenafil in retinopathy.

Retinopathy			
Preclinical Studies			
Animals	Animal Model	Dosage	Results
C57/B16N mouse pups	Oxygen-induced retinopathy	3 mg/kg bw, s.c.	Reduced retinal vascular obliteration and neovascularisation due to the stabilisation of HIF-1α expression during exposure to hyperoxia [130].
Sprague–Dawley rats	Streptozotocin-induced diabetic retinopathy	1 and 2.5 mg/kg bw, orally	Reduced ocular VEGF levels in diabetic rats [124].
Wistar rats	N-nitro-L-arginine methyl ester-induced hypertensive retinopathy	0.5 mg/kg bw, i.p.	Inhibited the progression of ischaemic injury and alterations in retinal vascular morphology [131].
Sprague–Dawley rats	Oxygen-induced retinopathy	50 mg/kg bw, orally	Significantly reduced the thickness of the outer plexiform layer [132].

bw, body weight; HIF-1α, hypoxia-inducible factor 1-alpha; s.c., subcutaneous; VEGF, vascular endothelial growth factor.

**Table 6:** Preclinical and clinical studies that evaluated the benefits of sildenafil in cancer.

Cancer		
In Vitro and Animal Studies		
Cancer Model	Dosage	Results
Prostate cancer PC-3 and DU145 prostate cancer cells	Sildenafil 10 μM + Doxorubicin 1.5 μM (PC-3) or 0.5 μM (DU145)	Enhanced doxorubicin-induced apoptosis by increasing ROS production, caspase-3, and caspase-9 activity and decreased Bcl-x expression and Bad phosphorylation [25].
Breast cancer 4T1 mammary carcinoma cells	Sildenafil 10, 30, 100 μM + Doxorubicin 1μM	The combination of sildenafil and doxorubicin had a synergistic effect inhibiting tumour cell growth [141].
Breast cancer MCF-7 breast cancer cells	5, 12.5, 25, 50 μg/mL	Determined the formation of necrotic tissue formation within the tumour and had cytotoxic effects [142].
Breast cancer MCF-7 breast cancer cells	Sildenafil 5, 12.5, 25, 50 μg/mL + cisplatin 5, 12.5, 25, 50 μg/mL	Enhanced the anticancer activity of cisplatin [142].
Neuroblastoma Human neuroblastoma cell line IMR-32	50 μM	Enhanced the development of neurite outgrowths that exhibited the expression of neuronal markers, including NeuN, NF-H, and βIII tubulin [143].
Breast cancer MCF-7 breast cancer cells	Sildenafil 40.33 μM + crizotinib 55.25 μM	The combination elicited significant apoptosis in breast cancer cells [144].
Colorectal cancer SW620, HT-29, HCT116, SW480, and SW1116 colorectal cancer cells	10 mM concentration	Suppressed cellular proliferation and the cell cycle and induced tumour cell apoptosis [145].
Lung cancer A549 human lung carcinoma cells	Sildenafil 15 μM + Doxorubicin 1.26 μM	The combination treatment resulted in tumour cell apoptosis through the reversal of ABC-transporter-mediated multidrug resistance and the downregulation of Nrf2 and ABCC1 [140].
Cervical cancer HeLa, HT-3, C33A, SiHa, U14 cervical cancer cells, and human cervical epithelial cells (HCerEpiC)	0.5, 1.0, 1.5 and 2.0 μM	Decreased cell viability and the expression of EMT marker proteins and p-Smad2/3 in HeLa cells was suppressed [23].
Prostate cancer Nude mice injected with PC-3 prostate cancer cells	Sildenafil 10 mg/kg bw, i.p. + Vincristine 0.5 mg/kg bw, i.p.	Enhanced vincristine-induced mitotic arrest and increased the susceptibility of apoptosis due to mitochondrial damage [146].
Prostate cancer BALB/cAnNCr-nu/nu male mice injected with PC-3 cells	Sildenafil 5 mg/kg bw, i.p. + Doxorubicin 1.5 mg/kg bw, i.p. or Sildenafil 10 mg/kg bw, orally + Doxorubicin 3 mg/kg bw, i.p.	Significantly inhibited tumour cell proliferation [25].
Breast cancer Balb/c female mice injected with 4T1 mammary carcinoma cells	Sildenafil 1 mg/kg bw i.p. + Doxorubicin 5 mg/kg bw, i.v.	The association treatment decreased tumour growth compared to the use of doxorubicin alone [141].
Breast cancer Albino female mice injected with Ehrlich ascites carcinoma cells	5 mg/kg, orally	Reduced tumour volume; decreased the levels of angiogenin, TNF-α, and the expression of vascular endothelial growth factor; and increased caspase-3 levels [142].
Breast cancer Albino female mice that received Ehrlich ascites carcinoma cells	Sildenafil 5 mg/kg bw, orally + Cisplatin 7.5 mg/kg bw, i.p.	Enhanced the anticancer activity of cisplatin [142].
Colorectal cancer Balb/c mice injected with SW480 or HCT116 colorectal cancer cells	50, 100 mg/kg bw, orally	Suppressed tumour growth [145].
Ascites tumour Swiss CD1 female mice injected with Ehrlich ascites carcinoma cells	1, 5 mg/kg bw, i.p.	Decreased the number of tumour cells; reduced their viability, growth rate, and ability to proliferate; and increased apoptosis [147].
Colorectal cancer Dextran-sulfate sodium (DSS)-induced colitis C57BI/6J male mice	5.7 mg/kg bw, in drinking water	The administration of sildenafil resulted in a 50% reduction in the number of colon polyps [148].
Malignant melanoma Mice expressing the human ret transgene in melanocytes	20 mg/kg bw, in drinking water	Decreased the levels of IL-1β, IL-6, VEGF, S100A9, and myeloid-derived suppressor cells. Reduced the immunosuppressive capabilities of myeloid-derived suppressor cells [149].
Clinical studies		
Type of cancer/Population	Dosage	Results
Prostate cancer Patients with ED	N/A	Reduced likelihood of receiving a diagnosis of prostate cancer [150].
Lymphangioma Phase 1, 2 study	10–20 mg, orally	Reduced both the volume and symptoms of lymphatic malformation in certain paediatric patients [151].
Breast cancer Phase 1 study	Sildenafil 100 mg, orally + Doxorubicin 75–360 mg/m^2^, i.v.	Did not provide cardioprotection after doxorubicin treatment [152].
Glioblastoma Brain cancer Phase 2 study	Sildenafil 50 mg + Sorafenib 400 mg + Valproic acid N/A orally	Enhanced the intracranial accumulation of anticancer agents and inhibited the proliferation of tumour cells by blocking the ABCG2 drug efflux pump within the BBB [153].

ABCC1, ATP-binding cassette sub-family C member 1; ABCG2, ATP-binding cassette, sub-family G, isoform 2 protein; Bad, BCL2 associated agonist of cell death; BBB, blood–brain barrier; Bcl-x, B-cell lymphoma-extralarge; bw, body weight; ED, erectile dysfunction; EMT, epithelial-to-mesenchymal transition; i.p., intraperitoneally; i.v., intravenous; IL-1β, interleukin 1ß; IL-6, interleukin 6; NeuN, neuronal nuclear protein; NF-H, heavy neurofilaments; Nrf2, nuclear factor erythroid 2-related factor 2; p-Smad2/3, phospho-SMAD2/SMAD3; ROS, reactive species of oxygen; S100A9, S100 calcium-binding protein A9; TNF-α, tumour necrosis factor α; VEGF, vascular endothelial growth factor A.

**Table 7:** Preclinical and clinical studies that evaluated the effects of sildenafil in depression.

Depression		
Preclinical Studies		
Population	Dosage	Results
Intruder-resident paradigm conducted on CD1 mice	10 mg/kg bw, i.p.	Enhances the levels of key neurotransmitters in the brain, specifically serotonin and noradrenaline, mitigating the exacerbation of depressive symptoms [161].
Swiss male mice with lipopolysaccharide-induced depression	5 mg/kg bw, i.p.	Reduced the duration of immobility observed during the forced swimming test. Increased the sucrose preference and levels of prepulse inhibition. Increased GSH levels and decreased lipid peroxidation and IL-1β levels [163].
Oxytocin receptor knockout mice	20 mg/kg bw, i.p.	Activation of oxytocin signalling pathways [162].
Male albino Swiss mice with restraint stress-induced depressive like behaviour	60 mg/kg bw, i.p.	Effectively restored the immobility generated by stress in the forced swim test [154].
Sprague–Dawley male rats with central muscarinic receptor blockade	Sildenafil 10 mg/kg bw, i.p. + Atropine 1 mg/kg bw, i.p.	The combination has notable antidepressant-like effects in the forced swim test. It decreases the density of cerebral β-adrenergic receptors [164].
Clinical studies		
ED associated with mild-to-moderate depressive disorder (n = 152)	25–100 mg, orally	Sildenafil significantly improved depressive symptoms and quality of life, lowering Hamilton depression scale scores [165].
ED associated with depression (n = 54)	N/A	Improved symptoms associated with depression, according to the Centre of Epidemiologic Studies—Depression Scale [166].
ED associated with depression in patients undergoing haemodialysis (n = 16)	N/A	Improved BDI scores compared to baseline [155].
ED associated with mild-to-moderate untreated depressive symptoms (n = 202)	25–100 mg, orally	Improved BDI II scores compared to the baseline measurements [156].
ED associated with depression and idiopathic Parkinson’s disease (n = 33)	50 mg, orally	Significantly reduced depression symptoms in 75% of the patients [160].

BDI, Beck Depression Inventory; bw, body weight; GSH, glutathione; i.p., intraperitoneally; IL-1β, interleukin 1β.

**Table 8:** Preclinical and clinical studies evaluating the effects of sildenafil in renal diseases.

Renal Disease		
Preclinical Studies		
Population	Dosage	Results
Streptozotocin-induced diabetic albino rats	3 mg/kg bw, orally	Enhanced kidney function by lowering oxidative stress; inhibiting pro-inflammatory HMGB1, TNF-α, MCP1, NF-kB, and IL1; and reducing caspase-3 levels [174].
Ioxilan-induced acute kidney injury in New Zealand white rabbits	6 mg/kg bw, orally	Reduced histological injury, acute kidney injury indicators (creatinine), and electrolyte disturbances (restores K^+^, Na^+^ levels) [178].
Iopromide-induced nephropathy in Wistar male rats	10 mg/kg bw, orally	Improved structural kidney damage, exhibiting a protective potential greater than that of N-acetyl cysteine [179].
Streptozotocin-induced diabetes in Sprague–Dawley male rats	3 mg/kg bw, in drinking water	Rats given sildenafil had a lower kidney-to-body weight ratio. Reduced urinary albumin excretion, renal cortical 8-OHdG levels, renal nitrotyrosine protein expression, and positive iNOS and ED-1 staining in glomeruli and tubule interstitium [180].
Streptozotocin-induced diabetic albino rats	Sildenafil 3 mg/kg bw, orally + Telmisartan 10 mg/kg bw, orally	Significantly reduced BUN, S.Cr, LDL, TGF-1, IL-1, proteinuria, and AGEPs while increasing SOD and NO [181].
Otsuka Long-Evans Tokushima fatty male rats	2.5 mg/kg bw, in drinking water	Significantly reduced albuminuria, glomerular hyperfiltration, glomerulosclerosis score, and the amount of nuclear antigen-positive glomerular and tubulointerstitial proliferating cells. Decreased collagen types I and III mRNA levels in the renal cortex [182].
Iohexol-induced nephropathy Wistar male rats	50 mg/kg bw, orally	Significantly reduced GFR and RBF, plasma creatinine, uraemia, and proteinuria [183].
Iohexol-induced nephropathy Wistar female rats	50 mg/kg bw, orally	Reduced serum and renal tissue levels of HIF-2α and sCr [184].
Streptozotocin-induced diabetic nephropathy in Sprague–Dawley rats	2.5 mg/kg bw, orally	Enhanced renal function by lowering triglyceride levels and increasing the number of podocytes [185].
Adenine-induced chronic kidney disease in Sprague–Dawley rats	0.1, 0.5, or 2.5 mg/kg bw, orally	Reduced the plasma concentration of the antioxidant indices in a dose-dependent manner. Reduced the levels of indoxyl sulfate, cytokine sclerostin, and neutrophil gelatinase- associated lipocalin [186].
Cisplatin-induced nephrotoxicity in Wistar male rats	0.4 mg/kg bw, i.p.	Decreased sCr, Bax/Bcl-2 ratio, caspase 3 expression, the number of TUNEL positive cells, and N-acetyl-b-D-glucosaminidase levels. Increased eNOS and iNOS activity and elevated renal blood flow [187].
Cisplatin-induced nephrotoxicity in Sprague–Dawley rats	2 mg/kg, i.p.	Decreased BUN, sCr, MDA, and TNF-α levels. Increased SOD levels and nitrite/nitrate concentrations [188].
Cyclosporine A-induced nephrotoxicity in Wistar male rats	5 mg/kg bw, orally	Reduced BUN, sCr, and MDA levels. Decreased urine albumin/Cr ratio, iNOS, TNF-α, and caspase 3 activity. Enhanced eNOS and GSH/NO/catalase activities [189].
Doxorubicin-induced nephrotoxicity in Sprague–Dawley rats	5 mg/kg bw, orally	Sildenafil reduced urea, sCr, uric acid, MDA, TNF-α, and caspase-3 levels, and raised GSH levels [190].
Ischaemia reperfusion-induced acute kidney injury in Wistar male rats	0.5, 1.0 mg/kg bw, i.p.	Increased creatinine clearance; decreased blood urea nitrogen and uric acid levels. Inhibited the elevation in thiobarbituric acid reactive substances and superoxide anion generation, while attenuating the decrease in GSH levels through the activation of PPAR-γ receptors [191].
5/6 nephrectomy in Wistar rats	5 mg/kg, orally	Effectively mitigated single nephron hyperfiltration and hypertension, inhibited the remodelling of renal arterioles, reduced systemic hypertension and proteinuria, enhanced the excretion of cGMP and nitrite/nitrate in urine, reduced oxidative stress, and ameliorated histological damage in the remaining kidney [192].
5/6 nephrectomy in Wistar rats	2.5 mg/kg bw, orally	Reduced sCr, systolic blood pressure, and proteinuria, while increasing urinary levels of nitric NO and cGMP [193].
Ischaemia-reperfusion renal injury In Sprague–Dawley rats	0.5 mg/kg bw, i.p.	Enhanced the recovery of renal injury by activating ERK, inducing the synthesis of iNOS and eNOS, and reducing the ratio of Bax to Bcl-2 [170].
Alloxan-induced diabetic nephropathy in Wistar male rats	3 mg/kg bw, orally	Reduced blood levels of urea and creatinine and decreased urinary albumin excretion. Increased levels of cGMP, antioxidant enzymes, and testosterone [194].
Deoxycorticosterone acetate-salt induced hypertension in Sprague–Dawley rats	50 mg/kg bw, orally	Reduced creatinine clearance and increased albumin-to-creatinine ratio. Reduced glomerulosclerosis and tubulointerstitial fibrosis. Inhibited the upregulation of ED-1, TGF-β1, and Bax, and the downregulation of Bcl-2 in the renal tissue [195].
Streptozotocin-induced diabetes in Sprague–Dawley rats	3 mg/kg bw, in drinking water	Decreased plasma concentrations of urea, creatinine, MDA, and NO. Increased GSH, Gpx, SOD, and CAT levels, as well as total antioxidant capacity [175].
Partial unilateral ureteral obstruction in Wistar rats	1 mg/kg bw, orally	Sildenafil exhibited a protective effect against tubular apoptosis [196].
Ischaemia-reperfusion renal injury Sprague–Dawley rats	1 mg/kg bw, orally	Reduced the levels of MDA, apoptotic cells, eNOS, and p53 positive cells [197].
Renovascular hypertension induced by the two-kidney-one-clip-operation in NO-GC1 KO mice	100 mg/kg bw, orally	Elevated cGMP levels, enhanced sensitivity to NO, and reduced systolic blood pressure [198].
Left renal artery clamping in C57BL/6 mice	40 mg/kg, orally	Reduced left and right kidney hypertrophy, as well as systolic blood pressure, heart rate, and intrarenal angiotensin I/II, while increasing plasma angiotensin 1–7 and NO levels [199].
Podocyte-specific deletion mice with streptozotocin-induced diabetes	5 mg/kg bw, orally	Reduced TRPC6 expression, glomerular desmin, urinary albumin, and increased nephrin [200].
Ischaemia-reperfusion renal injury in minipigs	0.7, 1.4 mg/kg bw, i.v.	Reduced systemic mean arterial pressure (1.4 mg/kg) and increased right ventricular function (0.7 mg/kg) [201].
Cardiopulmonary bypass in White Landrace crossbred female pigs	10 mg/kg bw, i.v.	Increased renal blood flow and NO production, while reducing proteinuria, IL-18 levels, cortical expression of endothelin-1, iNOS, and inflammatory cell infiltration. Prevented phenotypic alterations in proximal tubular cells [202].
Right–left nephrectomy White pigs	100 mg/kg bw, orally	Increased right ventricular function, NO levels, and decreased right ventricular resistance [203].
Right–left nephrectomy minipigs	100 mg/kg bw, orally	Increased right ventricular function, NO levels, and lowered renal vascular resistance. Reduced tubular oedema; improved endothelial cell integrity and mitochondrial ultrastructure [204].
Warm ischaemia in porcine kidneys	1.4 mg/kg bw, i.v.	Enhanced RBF and urine cGMP levels and lowered intrarenal resistance and sCr [172].
Folic acid induced acute renal injury in New Zealand white rabbits	0.3 mg/kg bw, i.p.	Upregulated the expression of COX1 and Tfam at mRNA level. Increased mtDNA copy number and downregulated KIM-1 levels [16].
Ischaemia-reperfusion renal injury in Mongrel dogs	1 mg/kg bw prior to operation, orally *OR* 0.5 mg/kg bw during the operation i.v.	Reduced sCr and BUN levels. Decreased the activity of caspase 3, TNF-α, IL-1β, and ICAM-1. Increased the expression of eNOS, GFR, and Nrf2 [205].
Clinical studies		
Population	Dosage	Results
PAH associated with impaired renal function (n = 277)	20, 40, 80 mg, orally	Decreased serum creatinine and increased glomerular filtration rate [176].
Patients with type 2 diabetes-associated microalbuminuria (n = 40)	50 mg, orally	Significantly reduced albuminuria and HbA1c levels [177].

8-OHdG, 8-hydroxy-2′–deoxyguanosine; AGEPs, advanced glycosylation end-products; Bax, B-cell lymphoma protein 2 (Bcl-2)-associated X; Bcl-2, B-cell lymphoma 2; BUN, blood urea nitrogen; bw, body weight; CAT, catalase; COX1, cyclo-oxygenase 1; eNOS, endothelial nitric oxide synthase; ERK, extracellular signal-regulated kinase; GFR, glomerular filtration rate; Gpx, glutathione peroxidase; GSH, glutathione; HbA1c, hemoglobin A1C; HIF-2α, heterodimeric nuclear transcription factor-2 alpha; HMGB1, high-mobility group box 1; i.p. intraperitoneally; i.v., intravenous; ICAM-1, intercellular adhesion molecule; IL-1, interleukin 1; IL-18, interleukin-18; IL-1ß, interleukin 1ß; iNOS, inducible nitric oxide synthase; KIM-1, kidney injury molecule-1; LDL, low-density lipoproteins; MCP1, monocyte chemoattractant protein-1; MDA, malondialdehyde; NO, nitric oxide; Nrf2, nuclear factor erythroid 2-related factor 2; RBF, renal blood flow; S.Cr, serum creatinine; SOD, superoxide dismutase; TGF-1, transforming growth factor-beta; TGF-β1, transforming growth factor-β1; TNF-α, tumour necrosis factor α; TRPC6, transient receptor potential cation channel 6.

**Table 9:** Preclinical and clinical studies evaluating the effects of sildenafil in gastrointestinal diseases.

Gastrointestinal Diseases		
Preclinical Studies		
Animal Model	Dosage	Results/Reference
Ethanol-induced gastric damage in Wistar male rats	1 mg/kg bw, orally	Protected against stomach damage by activating the NO/cGMP/K(ATP) pathway [217].
Indomethacin-induced gastric mucosal damage in Wistar male rats	5, 10 mg/kg bw, orally	Protected the stomach mucosa against the aggressive impact of indomethacin by increasing NO levels and inhibiting lipid peroxidation [217].
Indomethacin-induced gastric ulcer in Sprague–Dawley female rats	50 mg/kg bw, orally	Protected the stomach mucosa, mitigating indomethacin-induced damage [218].
Acetic acid-induced gastric ulcer in Sprague–Dawley rats	5, 10 mg/kg bw, orally	Reduced inflammation and enhanced the healing response in the stomach mucosa [208].
Cysteamine-induced duodenal ulcer in Wistar male rats	25 mg/kg bw, orally	Reduced oxidative stress and ameliorated the lesions observed in the duodenal mucosa [219].
Indomethacin-induced gastric ulcer in Swiss male rats	5, 25, 50 mg/kg bw, orally	Exhibited a dose-dependent gastroprotective effect, decreasing ROS levels, enhancing NO and antioxidant enzymes levels, improving stomach cellular viability, and restoring variables associated with gastroprotection [220].
Indomethacin-induced gastric ulcer in Wistar male rats	50 mg/kg bw, orally	Significantly decreased gastric acid secretion, ulcer score, tissue MDA, and TNF-α levels, while increasing NO levels [221].
Indomethacin-induced gastric ulcer in Wistar male rats	Sildenafil 10 mg/kg bw, orally + ranitidine 50 mg/kg bw, orally	The combination exhibited antiapoptotic action on the stomach mucosa [222].
Acetic acid-induced colitis in Sprague–Dawley rats	5 mg/kg bw, s.c.	Resulted in the preservation of colon microarchitecture and a decrease in lipid peroxidation, MPO activity, TNF-α, and IL-1β levels; increased GSH levels [216].
Trinitrobenzenesulphonic acid-induced colitis in Sprague–Dawley rats	25 mg/kg bw, orally	Reduced the colonic levels of MDA, MPO, CL, and TNF-α, while concurrently increasing the amount of GSH [211].
Trinitrobenzene sulphonic acid-induced colitis in Wistar male rats	25 mg/kg bw, orally	Decreased tissue levels of TNF-α, ameliorating inflammation [223].
Trinitrobenzene sulphonic acid-induced colitis in Sprague–Dawley male rats	1 mg/kg bw, i.p.	Reduced the levels of indicators associated with colonic damage (TNF-α, IL-1β, MPO, and MDA) [224].
Indomethacin-induced intestinal ulceration in Sprague–Dawley male rats	3–20 mg/kg bw, orally	Dose-dependently reduced the severity of the lesions. Inhibited MPO activity, iNOS production, and bacterial invasion [225].

ATP, adenosine triphosphate; bw, body weight; cGMP, cyclic, guanosine monophosphate; CL, lucigenin chemiluminescence; GSH, glutathione; IL-1β, interleukin-1ß; MDA, malondialdehyde; MPO, myeloperoxidase; NO, nitric oxide; TNF-α, tumour necrosis factor α.

**Table 10:** Preclinical and clinical studies evaluating the effects of sildenafil in cardiovascular diseases.

Cardiovascular Diseases		
Preclinical Studies		
Population	Dosage	Results
Adult ischaemic cardiomyocytes derived from WT mice	1 μM	Significantly decreased the number of trypan blue-positive necrotic cells [236].
C57/BL6 mice exposed to global ischaemia followed by reperfusion (Langendorff mode)	0.1 μM	Increased the activity of Na^+^/K^+^-ATPase, promoting the reperfusion process [237].
Ischaemic hearts of Wistar rats	3 μM	Elevated cGMP levels and concomitantly reduced the extent of the infarct [238].
Ischaemic isolated hearts of Wistar rats (Langendorff mode)	10, 20, 50, and 200 nM	Enhanced the coronary flow at lower levels of coronary perfusion pressure across all doses [239].
Piglet model of cardiopulmonary bypass and arrest	10 nM	Restored ATP levels, enhanced energy charge, and modified release of hypoxanthine and inosine [240].
C57BL6/J mice with heart hypertrophy	100 mg/kg bw, orally	Improved both systolic and diastolic function, reducing cardiac hypertrophy and cardiomyocyte apoptosis [232].
Constriction-induced left ventricular pressure overload in C57BL/6J male mice	200 mg/kg bw, in soft chow	Inhibited ERK and calcineurin activity in both the right and left ventricles [241].
Transaortic constriction–induced left ventricular pressure overload in PKGIa LZM mice	200 mg/kg bw, in food	Significant inhibition of cardiac hypertrophy and left ventricular systolic dysfunction [242].
WT mice exposed to left atrial incision (Langendorff mode)	0.71 mg/kg bw, i.p.	Decreased the extent of myocardial infarction following an episode of ischaemia [236].
ICR mice exposed to global ischaemia followed by reperfusion	0.71 mg/kg bw, i.p	Decreased the extent of the infarct through the activation of mitoKCa and mitoKATP [243].
ICR mice exposed to ischaemia followed by reperfusion	0.7 mg/kg bw, i.p.	Reduced the extent of the infarct, increasing both iNOS and eNOS levels [244].
ICR mice exposed to descending coronary artery occlusion followed by reperfusion	0.7 mg/kg bw, i.p.	Elevated cardiac SIRT1 activity, leading to a reduction in the extent of the myocardial infarction [245].
ICR mice exposed to descending coronary artery occlusion	21 mg/kg bw, i.p.	Ensured the preservation of fractional shortening. Reduced the left ventricular end-diastolic dilatation, fibrosis, and apoptosis [246].
ICR mice subjected to myocardial infarction through the closure of the left anterior descending coronary artery	0.71 mg/kg bw, i.p.	Reduced ischaemic injury score. Increased the expression of eNOS and iNOS proteins and the ratio of Bcl-2 to Bax. Reduced apoptosis and the left ventricular end-diastolic diameter [247].
Dystrophin-deficient mice	80 mg/kg bw, orally	Mitigated impairments in heart function. Improved both the myocardial performance index and the ratio of early diastolic filling velocity to late diastolic filling velocity [248].
iNOS knockout and eNOS knockout C57BL6/J mice exposed to ischaemia followed by reperfusion	0.06 mg/kg bw injection into the LV lumen	The administration of sildenafil resulted in a notable decrease in the extent of myocardial infarction. This indicates that the acute cardioprotective effects of low-dose sildenafil are not reliant on eNOS, iNOS, or cGMP [249].
PGC1α^−/−^mice subjected to transverse aortic constriction-induced pressure overload	200 mg/kg bw, orally	Enhanced cardiac function and remodelling. Improved mitochondrial respiration and increased the expression of PGC1α mRNA in the myocardium [250].
Sprague–Dawley rats exposed to ischaemia followed by reperfusion	0.75 mg/kg bw, i.p.	Significantly reduced the size of infarcted regions [251].
Wistar rats exposed to heterotopic cardiac transplantation	0.7 mg/kg bw, i.v.	Enhanced both systolic and diastolic function of the myocardium following a three-hour period of arrest. Determined a notable translocation of CPK delta [252].
Wistar rats exposed to ischaemia and reperfusion	50 mg/kg bw, orally	Suppressed the significant elevation of MDA levels [253].
Sprague–Dawley rats exposed to left anterior descending coronary artery occlusion followed by reperfusion	0.7 mg/kg bw, i.v.	Increased the density of capillaries and arterioles, enhancing blood flow. Increased the expression of VEGF and Ang-1 at mRNA levels during the initial reperfusion period [254].
Mongrel dogs exposed to blockage of the anterior descending coronary artery	2 mg/kg bw, orally	Prolonged the QT interval, particularly in the presence of ischaemic conditions [233].
Piglets exposed to untreated ventricular fibrillation, which was afterwards followed by open-chest cardiopulmonary resuscitation	0.5 mg/kg, i.p.	The administration of sildenafil resulted in a shift towards aerobic metabolism in energy use. Increased the levels of ATP and ADP, while decreasing the levels of lactate in the myocardial tissue [255].
Pigs exposed to ventricular fibrillation and cardiopulmonary resuscitation	0.5 mg/kg bw, i.p.	Partially reduced the elevated levels of plasma Ang II and Ang (1–7). Increased the expression of eNOS, cGMP, and iNOS [256].
Cardiac arrest pigs	0.5 mg/kg bw, i.p.	Reduced the expression levels of miR-155-5p and miR-145-5p [257].
Fixed banding of the venous pulmonary confluent-induced postcapillary PH pigs	25–50 mg/kg bw, orally	Reduced apoptotic cells. Regulated gene expression decreasing oxidative stress and enhancing anti-inflammatory activity within the myocardium. Improved ventricular function [258].
New Zealand rabbits with left anterior descending artery occlusion	0.7 mg/kg bw, i.p.	Elicited both immediate and delayed protective effects against ischaemia-reperfusion injury by the activation of mitochondrial KATP channels [259].
New Zealand rabbits exposed to ischaemia through the blockage of coronary arteries.	0.71 mg/kg bw, i.v.	Reduced the size of the infarct [260].
Clinical studies		
Patients with chronic heart failure (n = 46)	50 mg, orally	Decreased pulmonary artery pressure and alleviated dyspnoea, while improving brachial artery flow-mediated dilatation and enhancing breathing during physical exertion [234].
Patients with coronary artery disease (n = 24)	100 mg, orally	Induced dilation of pericardial arteries while interfering with platelet activation [261].
Patients with left ventricular failure (n = 100)	50 mg, orally	Enhanced left ventricular ejection fraction, increased performance on the 6 min walking test, and positively impacted Doppler-derived variables related to left ventricular diastolic function [235].
Patients with left ventricular systolic dysfunction (NYHA II-IV) (n = 34)	25–75 mg, orally	Enhanced exercise capacity, decreasing pulmonary vascular resistance and increasing the cardiac output during exercise [262].
Patients with non-ischaemic diabetic cardiomyopathy (n = 59)	100 mg, orally	The administration of sildenafil was linked to an anti-remodelling impact, which subsequently led to enhanced cardiac kinetics [263].
Patients with heart failure and preserved ejection fraction (n = 216)	20, 60 mg, orally	There was no change in exercise capacity, clinical condition, or quality of life [264].
Patients with coronary artery disease (n = 144)	100 mg, orally	Reduced the time elapsed until the onset of angina and the overall duration of activity [265].
Patients with microvascular coronary dysfunction (n = 23)	100 mg, orally	Enhanced the coronary flow reserve [266].

ADP, adenosine diphosphate; ANG (1–7), angiotensin 1–7; ANG II, angiotensin II; ANG-1, angiotensin 1; ATP, adenosine triphosphate; Bax, B-cell lymphoma protein 2 (*Bcl*-2)-associated X; Bcl-2, B-cell lymphoma 2; bw, body weight; cGMP, cyclic, guanosine monophosphate; CPK, protein kinase C; eNOS, *endothelial nitric oxide synthase*; i.p., intraperitoneally; iNOS, inducible nitric oxide synthase; i.v., intravenous; KATP channel, *ATP*-sensitive potassium channel; MDA, malondialdehyde; miR-145-5p, microRNA-145-5p; miR-155-5p, microRNA-155-5p; mitoKATP, mitochondrial ATP-sensitive K^+^; mitoKCa, mitochondrial Ca^2+^-activated K^+^; mRNA, messenger RNA; PGC1α, Peroxisome proliferator-activated receptor-γ coactivator 1-α; RNA, *ribonucleic acid*; VEGF, vascular endothelial growth factor.

**Table 11:** Preclinical and clinical studies evaluating the effects of sildenafil in lung diseases.

Lung Diseases		
Preclinical Studies		
Population	Dosage	Results
Ovalbumin-sensitised BP2 mice (asthma model)	Sildenafil 20 mg/kg bw, + L-arginine 50 mg/kg bw, i.p.	Exacerbated airway hyper-responsiveness [268].
Wistar rat pups with neonatal hyperoxic lung injury	50 mg/kg bw, orally	Extended survival, elevated levels of pulmonary cGMP, diminished the pulmonary inflammatory response, decreased fibrin deposition and right ventricular hypertrophy, and promoted alveolarisation [269].
Neonatal Wistar rats exposed to hypoxia to induce bronchopulmonary dysplasia	50, 100 mg/kg bw, orally	Facilitated the restoration of lung function by stimulating the activation of HIF-α and promoting the overexpression of VEGF [270].
Acrolein-induced airway inflammation in Sprague–Dawley rats	25 mg/kg bw, orally	Reduced the production of TNF-α, inhibited leukocyte migration, and decreased mucus hypersecretion. Prevented epithelial hyperplasia and metaplasia [271].
Bleomycin-induced lung fibrosis Sprague–Dawley rats	10 mg/kg bw, s.c.	Reduced lung fibrosis by decreasing the expression of TNF-α and IL-1β [215].
Meconium-induced acute lung injury in newborn Wistar rats	25 mg/kg bw, orally	Preserved lung tissue and mitigated the inflammatory burst comparable to dexamethasone [272].
Repetitive lung gavage saline induced acute lung injury rabbits	1 mg/kg bw, i.p.	Decreased the migration of cells, specifically neutrophils. Reduced the release of TNF-α, IL-8, and IL-6, and decreased the levels of nitrite/nitrate, 3-nitrotyrosine, and MDA. Prevented lung oedema development and reduced protein content in BAL and death of epithelial cells [273].
Clinical studies		
Patients with cystic fibrosis (n = 19) Phase 2 study	20, 50 mg orally	Enhanced exercise capacity in individuals diagnosed with cystic fibrosis [274].
Patients with mild-to-moderate cystic fibrosis (n = 36) Phase 1, 2 study	20, 40 mg, orally	Decreased sputum elastase activity. Well-tolerated [275].
Patients with idiopathic pulmonary fibrosis (n = 29) Phase 2 study	20 mg, orally	The administration of sildenafil did not yield a statistically significant increase in the distance covered during the 6 min walk test, nor resulted in a significant decrease in the Borg dyspnoea index [276].
Patients with swimming-induced pulmonary oedema (n = 10)	50 mg, orally	Decreased the pulmonary vascular pressures without any negative impact on exercise hemodynamics [277].
Patients with pulmonary embolism (n = 20) Early phase 1 study	50 mg, orally	The administration of one oral dose of sildenafil did not provide any significant enhancement in cardiac index, but reduced systemic blood pressure [278].
Children with mild-to-moderate lung disease (n = 20)	1 mg/kg bw, orally	Enhanced the overall well-being and physical capabilities of children with cystic fibrosis, but also had a notable negative impact on lung function [279].
Patients with idiopathic pulmonary fibrosis and PAH (n = 14) Phase 2 study	20–50 mg, orally	Enhanced the 6 min walk distance [280].
Patients with advanced idiopathic pulmonary fibrosis (n = 247) Phase 2 study	Sildenafil 20 mg + Pirfenidone 801 mg, orally	The co-administration of sildenafil with pirfenidone did not yield any therapeutic advantage [281].
Patients with idiopathic pulmonary fibrosis (n = 274) Phase 3 study	Sildenafil 20 mg + Nintedanib 150 mg, orally	The combination of nintedanib and sildenafil did not yield a statistically significant advantage when compared to the administration of nintedanib alone [282].

BAL, bronchoalveolar lavage fluid; bw, body weight; cGMP, cyclic, guanosine monophosphate; i.p., intravenous; IL-1β, interleukin 1β; IL-6, interleukin 6; IL-8, interleukin 8; MDA, malondialdehyde; s.c., subcutaneous; TNF-α, tumour necrosis factor α; VEGF, vascular endothelial growth factor.

## Data Availability

All data generated or analysed during this study are included in this published article.
